# Frequent Down Regulation of the Tumor Suppressor Gene A20 in Multiple Myeloma

**DOI:** 10.1371/journal.pone.0123922

**Published:** 2015-04-09

**Authors:** Katharina Troppan, Sybille Hofer, Kerstin Wenzl, Markus Lassnig, Beata Pursche, Elisabeth Steinbauer, Marco Wiltgen, Barbara Zulus, Wilfried Renner, Christine Beham-Schmid, Alexander Deutsch, Peter Neumeister

**Affiliations:** 1 Division of Hematology, Department of Internal Medicine, Medical University of Graz, Graz, Austria; 2 Institute of Pathology, Medical University of Graz, Graz, Austria; 3 Institute for Medical Informatics, Statistics and Documentation, Medical University of Graz, Graz, Austria; 4 Division of Medical and Chemical Laboratory Diagnostics, Medical University of Graz, Graz, Austria; University Medical Center of the Johannes Gutenberg University of Mainz, GERMANY

## Abstract

Multiple myeloma (MM) is a malignant clonal expansion of plasma cells in the bone marrow and belongs to the mature B-cell neoplams. The pathogenesis of MM is associated with constitutive NF-κB activation. However, genetic alterations causing constitutive NF-κB activation are still incompletely understood. Since A20 (*TNFAIP3*) is a suppressor of the NF-κB pathway and is frequently inactivated in various lymphoid malignancies, we investigated the genetic and epigenetic properties of A20 in MM. In total, of 46 patient specimens analyzed, 3 single base pair exchanges, 2 synonymous mutations and one missense mutation were detected by direct sequencing. Gene copy number analysis revealed a reduced A20 gene copy number in 8 of 45 (17.7%) patients. Furthermore, immunohistochemical staining confirmed that A20 expression correlates with the reduction of A20 gene copy number. These data suggest that A20 contributes to tumor formation in a significant fraction of myeloma patients.

## Introduction

Multiple myeloma (MM) is a monoclonal tumor of bone marrow (BM) plasma cells, characterized by the presence of monoclonal immunoglobulins in blood and urine in a majority of cases, and is frequently associated with organ dysfunction [1]. MM represents 10–15% of hematopoietic neoplasms and is responsible for 20% of deaths among hematologic malignancies [2]. Molecular studies revealed the expression of constitutively active NF-kB in bone marrow aspirates in the majority of myeloma patients [3,4]. Additionally, the clinical use of bortezomib-a proteasome inhibitor known to inhibit NF-κB—in the treatment of myeloma, demonstrates the importance of the constitutive activation of the NF-κB-pathway in the pathogenesis of myeloma [5,6]. Although the identification of mutations in NF-κB regulators in 17% of MM patients shed some light on the mechanisms responsible for the constitutive NF-κB activation, other genetic alterations involved in this pathway are still incompletely understood [7–9].

*A20* (also called *TNFAIP3*), a ubiquitin-modifying enzyme acting as negative regulator of NF-κB, is inactivated through epigenetic silencing, deletion and/or somatic mutations in a significant portion of the ABC subtype of diffuse large B-cell lymphoma (DLBCL) [10], of extranodal marginal zone lymphoma of mucosa-associated lymphoid tissue (MALT lymphoma) [11–13] and of Hodgkin’s lymphoma [12,14]. Re-expression of *A20* in *A20* deficient cell lines resulted in suppression of cell growth, reduction of NF-κB target gene expression, higher cytotoxicity and induction of apoptosis, demonstrating that *A20* acts as tumor suppressor gene in these lymphomas [10,12,14]. Furthermore, A20 has been identified as a susceptibility gene also for multiple inflammatory diseases including systemic lupus erythematodes, rheumatoid arthritis, psoriasis, diabetes mellitus type 1, colitis, and coronary artery disease in type 2 diabetes [15–20]. Therein, single-nucleotide polymorphisms of A20 are suspected to reduce its potent anti-inflammatory function. In A20 knock-out mice a higher rate of spontaneous inflammations have been observed, providing again a link of reduced A20 expression and the development of autoimmune disease [19, 21].

Since the role of the tumor suppressor A20 in multiple myeloma has not been investigated yet we aimed to elucidate the functional/mutational properties of A20 in myeloma patients and to add knowledge in a promising target of anti-myeloma treatment.

## Material and Methods

### Patient samples, cell lines and DNA extraction

Fresh frozen BM material from 46 MM patients was used for this study. All samples were collected and stored at the Institute of Pathology at the Medical University Graz. Genomic DNA was isolated using the QIAamp® DNA Mini Kit (Qiagen; Hilden; Germany) according to the manufacturer’s instructions. The study was performed according to the Austrian Gene Technology Act and has been approved by the Ethical Committee of the Medical University Graz. For this retrospective study, we used patient specimens gained for routine diagnostic investigations, therefore, no written informed consent of patients was obtained. This consent procedure was approved by the Ethical Committee of the Medical University Graz.

Human B-cell lymphoma cell lines KM-H2 (DSMZ, Heidelberg, Germany) and UH3 cells (kindly provided by R. Dalla-Favera, Columbia University, New York, USA) were maintained in RPMI1640 (Invitrogen, Karlsruhe, Germany) media supplemented with 10% fetal calf serum (Invitrogen, Karlsruhe, Germany) and antibiotics (pen strep—Gibco) (Invitrogen, Karlsruhe, Germany). All cell lines were cultivated at 37°C and 5% CO2.

### Sequencing, methylation specific PCR, deletion analysis and mRNA gene expression

PCR products were purified and sequenced from both sides using the BigDye terminator chemistry 3.1 (Applied Biosystems, Foster City, CA, USA). Sequences were run on an ABI3130-xl automated sequencer (life technologies, Waltham, MA, USA). Sequences were confirmed by 2 independent PCR reactions.

For methylational analysis genomic DNA of MM cases were treated with CT conversion reagent (MethylCode Bisulfite Conversion Kit, life technologies, Waltham, MA, USA) according to the manufactures’ protocol. Two independent PCR reactions were performed to detect either methylated or unmethylated CpG island of A20. Each PCR product was directly loaded onto a nondenaturating 3% agarose gel, stained with ethidium bromide and directly analyzed. CpGenomeTM Universal Unmethylated DNA (Chemicon International, Billerica, MA, USA) as methylation negative control and CpGenomeTM Universal Methylated DNA (Chemicon International, Billerica, MA, USA) as positive control were included.

Copy number assays were performed on genomic DNA of MM samples in triplicates RQ-PCR using an ABI Prism 7000 Detection system (Applied Biosystems, Foster City, CA, USA) and SYBR®Green PCR Master Mix (Invitrogen, Waltham, MA, USA). *RPPH* and *TERT* served as endogenous controls. The ΔΔCT was used to measure the relative copy number as described by Aarskog et al. [22].

Total RNA was extracted using the Trizol (Invitrogen) according to the manufacturer’s protocol. cDNA was synthesized using the RevertAid™ H Minus First Strand cDNA Synthesis Kit (Fermentas, Waltham, MA, USA). The following commercial available Realtime PCR assays (Applied Biosystems, Invitrogen, Carlsbad, CA) were used for real time RT-PCR: A20 (Hs00234713_m1), BCL2 (Hs00608023_m1), CCND (Hs00608023_m1), CCR7 (Hs01013469_m1), CD44 (Hs01075861_m1), CXCR2 (Hs01891184_s1), FLIP (Hs00153439_m1) and IRF-4 (Hs01056533_m1). PCR reactions were performed using an ABI Prism 7000 Detection system (Applied Biosystems, Invitrogen, Carlsbad, CA). GAPDH (commercial assay: Hs02758991_g1, Applied Biosystems, Invitrogen, Carlsbad, CA), PPIA (commercial assay: Hs04194521_s1, Applied Biosystems, Invitrogen, Carlsbad, CA), and HPRT1 (commercial assay: Hs02800695_m1, Applied Biosystems, Invitrogen, Carlsbad, CA), which are known to exhibit the lowest variability among lymphoid malignancies served as housekeeping genes [23]. The results are expressed as relative units based on calculation 2^-ΔΔCT^, which gives the relative amount of target gene normalized to the endogenous control (geometric mean of the two house keeping genes) and relative to a normalized sample.

The nucleotide acid sequences for the primers for these purposes are shown in the S1 Table.

### Immunohistochemical analysis of A20

Frozen section was stained using the UltraVision LP HRP Polymer detection system (ThermoFisher, Fremont, CA USA). Primary antibody to A20 (*TNFAIP3)* was purchased (ab92324, dilution 1:10;Abcam, Cambridge, United Kingdom). For control purposes, tissues (lymph node metastases of breast carcinoma) known to contain the respective antigens were included. Replacement of the primary antibody by normal serum always revealed negative results. Additionally, to gain knowledge on the A20 expression in lymphoid cell, we perform immunohistochemical analysis on normal tonsil on normal bone marrow. Scoring of tissue slides and determination of the immunoreactive score (IRS) was performed as previously described [24].

### Structural analysis of the zinc finger 7 domain

Structural information about A20 was available in the PDB structure database, an international repository for 3-D structure files [25]. In this paper we used the 3VUW18 and the 3DKB19 data file. From the PDB database these coordinate files can be downloaded via internet, and the protein visualized at the local place. The visualization of the A20 protein was done with the Swiss-Pdb viewer20 [26]. The electron behavior in molecules was treated by methods based on quantum theoretical calculations. Quantum theoretical calculations aid the exploration of processes that provide the link between structural analysis and physicochemical and biological functions [27,28].

### Statistical Analyses

All statistical analyses were performed using the Statistical Package for Social Sciences version 17.0 (IBM, NY, USA) as previously described [24]. Briefly, the non-parametric Kruskal—Wallis test was used to analyse differences in the expression levels among normal bone marrow and MM with/without monoallelic A20 deletion. The expression levels with significant differences in their expression were analysed using the Mann—Whitney U-test; all significant associations were further corrected for multiple testing by applying a Bonferroni correction, dividing the significance level by the number of variables tested. The Spearman correlation test was performed to examine any correlation of the A20-mRNA and-gene copy number levels to immunohistochemistry of A20. When the p value was lower than 0.05, a significant value was reached. Expression levels are presented as mean values ± standard deviation (SD). All statistical tests were 2- sided.

## Results

### A20 is mutated and exhibited a reduced copy number in multiple myeloma

**Fig 1:**
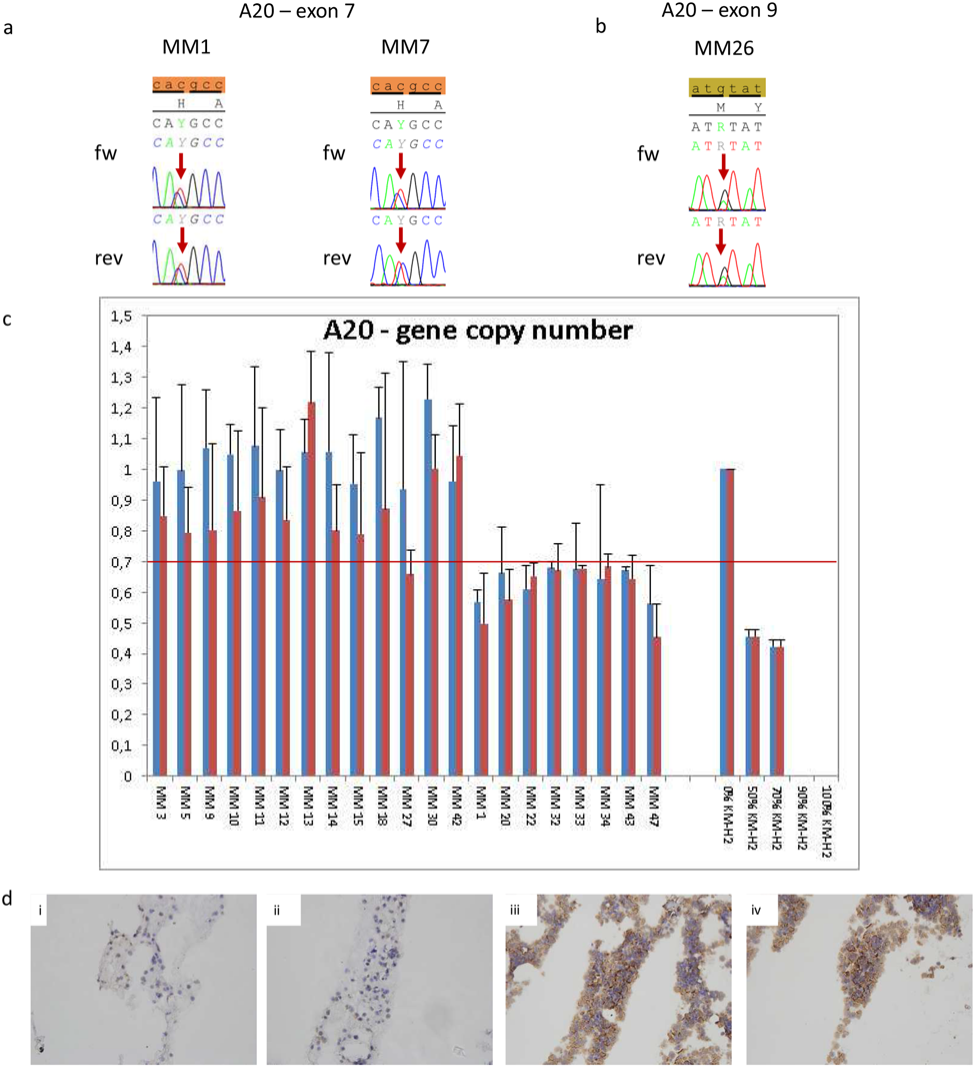
Genetic aberrations of A20 in multiple myeloma. **a: Electropherogram of rs368271377 in exon 7:** Arrows indicate the single base pair substitution. **b: Electropherogram of rs143002189 in exon 9:** The arrow indicates the single base pair substitution. **c: Gene copy number analysis of A20 of selected cases:** For the gene copy number assays two technical replicates of each samples were used. The blue bar represents the data for exon 4 and the red one for exon 6. Each bar represents the mean values of expression levels ± standard deviation (SD). Cut off for deletion—depicted as red line—was set at 0.7 through the fact that samples exhibited up to 40% non-neoplastic surrounding tissue. **d: Representative immunohistochemical A20 staining of multiple myeloma samples.** i and ii: multiple myeloma samples with reduced A20 gene copy number. iii and iv: multiple myeloma samples with normal A20 gene copy number.

In total, 46 myeloma patient specimens were analyzed by direct sequencing. A20 was heterozygously mutated in three (6.5%) of 46 myeloma specimens (Table 1). One silent single base pair substitution –c.1524C>T (His508, rs368271377)—was found in two patients (Fig 1a). In a third patient, a missense single base pair substitution- c.2364G>A (Met788Ile, rs143002189)—was detected (Fig 1a). Furthermore, we sequenced the corresponding normal tissue of the three cases exhibiting the silent and missense single base pair substitution and demonstrated their germline origin. In addition, we searched the NCBI SNP database for the TNFAIP3 variations discovered. For the silent His508His (rs368271377) exchange, no frequency data was available. The Met788Ile (rs143002189) exchange was detected in 1 out of 4370 chromosomes, resulting in minor allele frequency lower than 0.001.

**Table 1:** Summary of clinical data and A20 mutations, gene copy numbers and expression of the analysed MM patients.

ID	Subtype	Cytogenetics	A20 sequence analysis	A20 gene copy number	A20 expression
MM1	polymorphic	t11/14	rs368271377	reduced	low
MM2	polymorphic	del17, del13		normal	high
MM3	lymphocytic	t11/14		normal	moderate
MM4	lymphocytic	n.a.		normal	high
MM5	plasmablastic	del13		normal	n.d.
MM6	plasmablastic	normal		normal	high
MM7	polymorphic	normal	rs368271377	normal	low
MM8	lymphocytic	normal		normal	moderate
MM9	lymphocytic	t11/14		normal	high
MM10	plasmablastic	del13		normal	n.d.
MM11	polymorphic	hyperploid		normal	n.d.
MM12	lymphocytic	n.a.		normal	high
MM13	lymphocytic	n.a.		normal	moderate
MM14	polymorphic	n.a.		normal	high
MM15	plasmablastic	normal		normal	moderate
MM16	polymorphic	n.a.		normal	n.d.
MM17	polymorphic	n.a.		normal	high
MM18	plasmablastic	del13		normal	moderate
MM19	lymphocytic	n.a.		normal	n.d.
MM20	polymorphic	n.a.		reduced	low
MM21	polymorphic	n.a.		normal	n.d.
MM22	polymorphic	trisomy11, del13		reduced	low
MM23	lymphocytic	n.a.		normal	low
MM24	polymorphic	del13		normal	moderate
MM25	lymphocytic	t11/14		normal	low
MM26	polymorphic	n.a.	rs143002189	normal	n.d.
MM27	polymorphic	del13, monosomy14, trisomy9		normal	low
**ID**	**Subtype**	**Cytogenetics**	**A20 sequence analysis**	**A20 gene copy number**	**A20 expression**
MM28	plasmablastic	n.a.		normal	n.d.
MM29	plasmablastic	del13		normal	moderate
MM30	lymphocytic	n.a.		normal	high
MM31	lymphocytic	del13		normal	high
MM32	polymorphic	normal		reduced	low
MM33	lymphocytic	n.a.		normal	low
MM34	polymorphic	n.a.		reduced	n.d.
MM36	polymorphic	del13, trisomy 4, trisomy 9		normal	moderate
MM37	plasmablastic	n.a.		normal	n.d.
MM38	plasmablastic	del13		normal	moderate
MM40	polymorphic	n.a.		normal	high
MM41	lymphocytic	normal		normal	high
MM42	lymphocytic	del13		normal	high
MM43	polymorphic	del13		reduced	low
MM44	lymphocytic	n.a.		n.d.	n.d.
MM46	polymorphic	n.a.		normal	moderate
MM47	polymorphic	del13, trisomy 9		reduced	low
MM48	lymphocytic	n.a.		normal	high
MM49	lymphocytic	n.a.		normal	moderate

n.a. denotes not available

n.d. denotes not done

**Fig 2:**
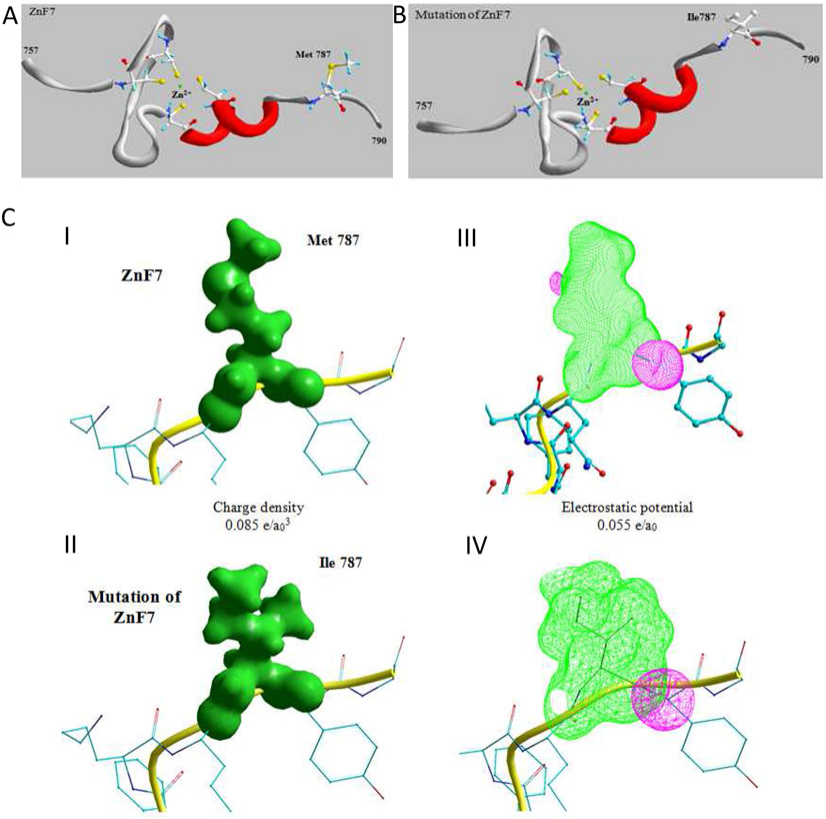
Structural analysis of A20 with and without rs143002189. a) The methionine (Met) residue at position 788 in the amino acid chain of the zinc finger domain 7 (ZnF7) has been changed to isoleucine. b) Shows the different charge density distributions of the unmutated- (I) and the mutated ZnF7 (II). Surfaces with constant density values: 0.085 e/a_0_^3^ are shown. III and IV show the corresponding spatial distribution of the electrostatic potential surrounding the molecule. Positive values are red encoded, negative values green. The surface shows constant values of the electrostatic potential with: 0.055 e/a_0_.

To study putative structural effects of the rs143002189 single base pair substitution in the zinc finger domain 7 (ZnF7), the affected residues at position 788 (methionine (Met) → isoleucine (Ile)) inside the domain were interchanged (Fig 2a). Methionine and isoleucine are neutral, hydrophobic and aliphatic. Methionine contains a sulfur atom. The force field energy was = -772KJ/mol for the non-mutated domain and -751KJ/mol for the mutated domain. Additionally, the electron density (Fig 2BI and 2BII) and the electrostatic potential (Fig 2BIII and 2BIV) were altered by the rare germline exchanges in ZnF7. Together, this rare germline exchange results in a conformational change and as a consequence might result in a reduced steric interaction and a possibly reduced A20 protein function.

To investigate whether the A20 gene was deleted in myeloma patients, RQ- PCR gene copy assays of exon 4 and exon 6 of the A20 gene were performed in 45 MM specimens. For testing the sensitivity and specificity of the two designed A20 gene copy number assays being located in exon 4 and exon 6, two cell lines, KM-H2—knowing to be deleted in exon 3 to exon 6 [12] and UH3 without any A20 deletion, were mixed together in in a serial dilution (0%, 50%, 70%, 90% and 100% of KM-H2 cells). Percentage of KM-H2 cells correlated (Spearman-rho: -0.980, p<0.01 for each RQ-PCR assays; Fig 1b) with the A20 content demonstrating an excellent specificity and sensitivity for our designed RQ-PCR assay. The RQ-PCR reaction were repeated independently, the mean values for each sample were calculated and used for further analysis. Due to the fact that all MM specimens exhibited up to 40% non-neoplastic surrounding tissue, 2^-ΔΔCT^ values of all specimens below 0.7 were defined as deleted. Eight out of 45 MM (17.7%) specimens exhibited monoallelic deletions in the A20 locus (Fig 1b). To determine a possible causal relationship between diminished A20 and the clinical course of disease we correlated the gene copy number with mutational or cytogenetic alterations as well as with other prognostic parameters like myeloma subtype or International Staging System. However, no correlation between these parameters could be identified in our cohort.

To further analyze whether the A20 expression was altered through promoter hypermethylation we performed methylation specific PCR, but could not find any evidence for promoter methylation of CpG-islands in any of the 46 MM specimens (data not shown).

### Reduced A20 gene copy number resulted in diminished A20 protein expression in multiple myeloma

**Fig 3:**
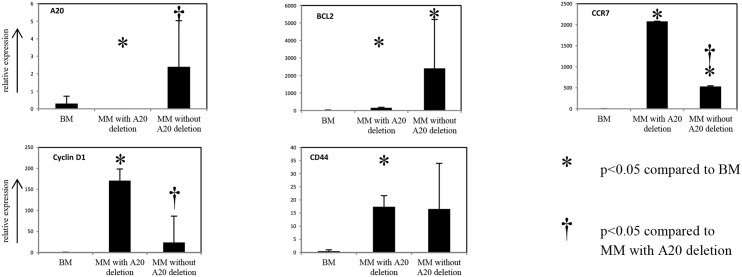
mRNA expression analysis of A20 and 7 NF-κB target genes (BCL2, Cyclin D1, CCR7, CD44, CXCR2, cFlip, IRF4) of MM cases with (n = 6) and without (n = 14) monoallelic A20 deletions and of non-neoplastic bone marrow biopsies (BM; n = 6). mRNA expression levels were calculated as relative expression in comparison with peripheral mononucleated cells serving as a calibrator. Each bar represents the mean values of expression levels ± standard deviation (SD). The comparison of the expression levels was performed by using the Mann-Whitney U test; all significant associations were corrected for multiple testing by applying a Bonferroni correction.

To clarify whether reduced A20 gene copy number resulted in diminished A20 protein expression, immunohistochemical analysis was performed in 35 myeloma specimens. 13 myeloma patients showed a distinct positivity for A20 in the majority of MM cells (>50%, high expression). In 11 cases, an A20 expression was only detected in the minority of MM cells (20–50%, moderate expression) and in another 11 cases the positivity for A20 was lower than 10% of myeloma cells or even lacking (low expression). Six of the eight MM specimens exhibiting the monoallelic A20 deletion, were processed for immunohistochemical analysis, and consequently all of them A20 expression of myeloma cells occurred in fewer than 10%. By correlating IRS to A20 gene copy number a positive correlation of A20 expression to the presence of monoallelic A20 deletions was observed (Spearman rho = -0.454, p<0.01, Fig 1c). Additionally, A20 mRNA expression analysis of selected cases (6 MM cases with monoallelic A20 deletion and 14 MM cases without monoallelic A20 deletions) demonstrating a significant correlation between the presence of monoallelic A20 deletions (Spearman rho = -0.704, p<0.01) and the IRS of A20 IHC analysis (Spearman rho = 0.86, p<0.01, Fig 3). Taking together these data suggest that monoallelic deletion causes reduced A20 expression in patients with MM.

Furthermore, mRNA expression analysis of A20 and 7 NF-κB target genes (BCL2, Cyclin D1, CCR7, CD44, CXCR2, cFlip, IRF4) [29] of MM cases with (n = 6) and without (n = 14) monoallelic A20 deletions and of non-neoplastic bone marrow biopsies (BM; n = 6) was performed. MM exhibiting monoallelic A20 deletion showed a 723 fold (p<0.01) and 5800 fold (p<0.01, Fig 3) lower expression of A20 compared to BM and MM without A20 deletions. Two of the 7 NF-κB target genes—namely BCL2 and CCR7- were at least 17 fold higher expressed in MM cases with and without A20 deletion (p<0.02, Fig 3) whereas Cyclin D1 and CD44 were at least 35 fold higher expressed in MM cases with A20 deletions (p<0.01, Fig 3) compared to BM. Interestingly, CCR7 and Cyclin D1 were at least 4 fold higher expressed in MM cases exhibiting A20 deletions compared to MM cases without A20 deletions (p<0.01, Fig 3). Therefore, we suggest that A20 deletions exert some functional consequences on NF-κB signaling

## Discussion

This study was designed to investigate the genetic and epigenetic status of A20 in myeloma patients. A20, a negative regulator for the NF-κB-pathway, has been shown to be inactivated through somatic mutations, deletions and/or promoter hypermethylation in various B-cell malignancies, causing constitutive NF-κB activation [10,13,30–35]. NF-κB deregulation was also found to be constitutively activated in MM [3,4]. However, activating mutations in positive regulators and inactivating mutations in negative regulators of this pathway have only been identified in 20% of MM [36]. We could identify two synonymous, silent mutations (c.1524C>T) and one missense mutation (c.2364G>A) leading to an amino-acid exchange and coding of isoleucine instead of methionine. The c.2364G>A mutation was found be to be of germline origin. Germline A20 functional abnormalities were also detected 77% of patients with primary Sjögren’s syndrome and MALT lymphoma and play a key role in lymphomagenesis in the context of autoimmunity [37]. Thus, it might be speculated that A20 germline mutations represent a predisposition to lymphoma development. The mutation c.2364G>A mutation was found to be located within exon 9, the coding region for the ZnF7 of the A20 protein, where A20 ligase activity is assumedly located. Due to the substitution of the aliphatic, nonpolar aminoacid methionine by isoleucine generating a more compact appearance, a diminished steric interaction leading to a reduced A20 protein function might be the consequence. In our cohort of MM samples, we detected a reduced A20 gene copy number in 17.7%. In a large cohort of 120 MM samples with abnormal karyotype, del6, where TNFAIP3 is located, was found in 33% of patients sample [38]. Furthermore, reduction of A20 gene copy number was associated with diminished A20 expression as demonstrated by immunohistochemistry. However, since we also detected low A20 protein expression in myeloma specimens without a reduced gene copy number, it seems that additional, so far unidentified mechanisms are responsible for the down-regulation of A20 in these patients. Global mRNA gene expression profiling on purified CD138 plasma cells of 320 newly diagnosed myeloma patients identified a novel NF-κB cluster resulting in constitutive activation of the noncanonical NF-κB pathway with a significant better response to bortezomib [39]. Therefore, it is plausible that A20 inactivation by deletions significantly contribute to the pathogenesis of myeloma in a significant fraction of patients.

A further indication for the impact of A20 in the NF-κB pathway could be demonstrated by investigating A20 expression and the expression of 7 NF-κB target genes in MM cases with and without monoallelic A20 deletions and of non-neoplastic bone marrow biopsies. As expected, A20 expression was lower in MM samples with monoallelic A20 deletion. Interestingly, CCR7 and Cyclin D1 were higher expressed in MM cases exhibiting A20 deletions compared to MM cases without A20 deletions, demonstrating a possible effect on the NF-κB signaling cascade.

Promoter methylation was reported to be responsible for the inactivation of A20 in various lymphoma entities, especially [40] in activated B-cell like DLBCL and mantle cell lymphoma, [33] where an inverse correlation of methylation and expression levels was found in these two entities. However, in our study no evidence of A20 inactivation through promoter methylation could be found.

In conclusion we detected an A20 inactivation through deletions, rather than somatic mutations or promoter methylation. Through constitutive NF-κB activation, this inactivation could promote the pathogenesis of MM.

These findings might significantly contribute to the understanding of the molecular events involved in myeloma development and defines A20 as potential therapeutical target for the future anti myeloma therapy.

## Supporting Information

S1 Table Primer sequences. (DOCX)
