# Supplementary material for: Frequent Down Regulation of the Tumor Suppressor Gene A20 in Multiple Myeloma
Source: PLoS One. 2015 Apr 9;10(4):e0123922. doi: 10.1371/journal.pone.0123922 (PMC4391781; doi:10.1371/journal.pone.0123922)
Supplement: S1 Table — (DOCX) [file pone.0123922.s001.docx]

Supplementary Table S1: Primer sequences

| **Gene - Primer ID** | **Sequence** | **Application*** |
| --- | --- | --- |
| Exon 2 forward | 5’ CCGGGAGTAGAGGTGCTA 3‘ | SA |
| Exon 2 reverse | 5‘ GTCTGCTATTATCACATACCCC 3‘ | SA |
| Exon 3 forward | 5‘ TCAGTTTGCCCTTGACTAGGA 3‘ | SA |
| Exon 3 reverse | 5‘ TGAGTCCCACTGGAGGTTTC 3‘ | SA |
| Exon 4 forward | 5‘ GGGAGTACAGGATACATT 3‘ | SA |
| Exon 4 reverse | 5‘ GCTGGAAAGCATTTAAGTA 3‘ | SA |
| Exon 5 forward | 5‘ ACCTAAGGGCCTCATTTTCC 3‘ | SA |
| Exon 5 reverse | 5‘ AGCAAAAAGGAAAACCGTGA3‘ | SA |
| Exon 6 forward | 5‘ TGAGATCTACTTACCTATGGCCTTG 3‘ | SA |
| Exon 6 reverse | 5‘ AGGGTGGCAGCAACTCAG 3‘ | SA |
| Exon 7 forward | 5‘ GCTAATGATGTAAAATCTTGTGTGTG3‘ | SA |
| Exon 7 reverse | 5‘ CAGGAACAAAACCCCTTCTG3‘ | SA |
| Exon 8 forward | 5‘ CTCTGTATCGGTGGGGTGAC3‘ | SA |
| Exon 8 reverse | 5‘ CAAAAAGCATCGAACACACG3‘ | SA |
| Exon 9 forward | 5‘ GCTTGGCGGTTTTCCTCAG3‘ | SA |
| Exon 9 reverse | 5‘ CTTTGCTTTCTAAGGCCACCT3‘ | SA |
| Exon 4 forward | 5‘ TCAGTACAACTCACTGGAAGAAATACAC3‘ | qPCR |
| Exon 4 reverse | 5‘ AGGATGTTGCAAAGGACAAATATG3‘ | qPCR |
| Exon 6 forward | 5‘ AATCCGAGCTGTTCCACTTGTT 3‘ | qPCR |
| Exon 6 reverse | 5‘ AAGTCTTCAAATCTTCCCCGGT 3‘ | qPCR |
|  |  |  |
|  |  |  |
| **Gene - Primer ID** | **Sequence** | **Application** |
| TERT forward | 5‘ GGGAAGCATGCCAAGCTCT 3‘ | qPCR |
| TERT reverse | 5‘ CACGCTCATCTTCCACGTCA 3‘ | qPCR |
| RPPH1 forward | 5‘ CTTTGCCGGAGCTTGGAAC 3‘ | qPCR |
| RPPH1 reverse | 5‘ GCCATTGAACTCACTTCGCTG 3‘ | qPCR |
| A20_meth_fw | 5‘ TTTTCGGAGAGGTAATCGTC 3‘ | MSP |
| A20_meth_rv | 5‘ AACGCCAAATAAACGATACC 3‘ | MSP |
| A20_meth_fw | 5‘ TTGGCGTTTTGTTTTTTTC 3‘ | MSP |
| A20_meth_rv | 5‘ CGTCGAACTAATCCTACACAA 3‘ | MSP |
| A20_unmeth_fw | 5‘ GTTTTTTGGAGAGGTAATTGTT 3‘ | MSP |
| A20_unmeth_rv | 5‘ CCAACACCAAATAAACAATACC 3‘ | MSP |
| A20_unmeth_fw | 5‘ GTTGGTGTTTTGTTTTTTTT 3‟‘ | MSP |
| A20_unmeth_rv | 5‘ CCATCAAACTAATCCTACACAA 3‘ | MSP |

SA denotes direct sequencing

qPCR denotes gene copy number assay

MSP denotes methylation specific PCR
